# Enhancing student nurses’ clinical education in aged care homes: a qualitative study of challenges perceived by faculty staff

**DOI:** 10.1186/s12912-021-00632-0

**Published:** 2021-06-26

**Authors:** Kristin Laugaland, Stephen Billett, Kristin Akerjordet, Christina Frøiland, Laurie Grealish, Ingunn Aase

**Affiliations:** 1 SHARE- Centre for Resilience in Healthcare, Faculty of Health Sciences, University of Stavanger, Kjell Arholms gate 41, 4036 Stavanger, Norway; 2 Education and Professional Studies, Griffith University, Brisbane, Australia; 3 Menzies Health Institute Queensland, Griffith University and Gold Coast Health, Gold Coast, Australia

**Keywords:** Clinical education, First-year student nurses, Aged care home placements, Challenges, Perceptions, Experiences, Faculty staff

## Abstract

**Background:**

Ageing populations are increasing the demand for geriatric care services. As nursing schools respond to this demand, more high-quality clinical placements are required, and aged care homes offer suitable placement sites. Although an aged care experience for students is beneficial, the basis for effective implementation of these placements is yet to be fully established. The aim of this study was to explore faculty staff perspectives on the challenges associated with providing effective clinical education in aged care homes for first-year student nurses.

**Methods:**

An exploratory qualitative study was performed. Fifteen in-depth interviews were conducted with program leaders of nursing degree programs (*n* = 4), course leaders (*n* = 6) and practice coordinators (*n* = 5) in three Norwegian universities. Data were analysed using thematic analysis. The findings were reported using the Standards for Reporting Qualitative Research (SRQR).

**Results:**

Five themes were identified regarding the perceived challenges to implementing effective clinical education in aged care homes: (1) low staffing levels of registered nurses limit the capacity to effectively host students; (2) prevalence of part-time teachers can compromise the quality of students’ learning experiences; (3) tensions about the required qualifications and competencies of nurse teachers; (4) variation in learning assessments; and (5) lack of quality assurance.

**Conclusions:**

These challenges signal key areas to be addressed in quality assurance for effective aged care placements. Further research into the minimum staffing levels required to support student learning in the aged care setting is required. Methods for developing shared practices to facilitate learning in aged care homes need to address the prevalence of part-time teaching appointments. Further research into the levels of qualification and competence required to support student learning in aged care facilities can assist with setting standards for this sector. Finally, academic-practice institutions must engage with government officials and national nursing bodies to develop national standards for clinical education in aged care homes.

**Supplementary Information:**

The online version contains supplementary material available at 10.1186/s12912-021-00632-0.

## Background

Populations are ageing globally, with one in six people expected to be over 65 years of age by 2050. In 2018, for the first time in history, people aged 65 years and older outnumbered children under five years of age [1]. Further, the number of people aged 80 years and older is expected to triple by 2050 [1], making the ageing of the population a momentous demographic transformation in the 21st century.

Nurses constitute a significant percentage of the health and social workforce, so university nursing programs are essential in preparing a workforce to meet future healthcare needs, including those associated with an ageing population [2]. However, a scoping review found little evidence of geriatric or gerontologic theory components in nursing education [3]. Instead, the evidence suggests that nursing students mostly learn about ageing and aged care through placements in aged care homes [4].

The renewed interest in aged care homes as clinical placement settings for nursing students has been prompted by the growing health care needs of an ageing population, saturated clinical placements in health services, and regulatory requirements in countries such as Norway [4]. Aged care home placements are often provided for first-year students to foster learning about the essence of nursing [5] and instil positive attitudes about older people [6].

A review of nursing students’ experiences of aged care home placements found that students valued the chance to build relationships with older people, improve their communication skills, and assist the elderly with their daily living activities, but noted that students had to overcome several challenges to achieve learning [2]. For example, when assigned to aged care homes for clinical placement, nursing students did not value working with personal care assistants and often reported that the practice sites were unprepared for them to learn effectively [2].

A recent scoping review of teaching strategies and activities to enhance students’ clinical placement in residential aged care facilities found that the most common strategy was to use care staff to provide facility orientation and to serve as student mentors [7]. Whilst this review highlighted a range of strategies, the effectiveness of these strategies was not addressed. The researchers identified timing and delivery of teaching strategies and the need for a multi-faceted and collaborative approaches as critical to student placements in residential aged care facilities [7]. However, methods to deliver strategies remains problematic in this setting.

A review of nurse staffing standards in aged care homes across six countries found a reliance on personal care workers rather than professional or registered nursing staff [8]. In Norwegian nursing homes, 20 % of nursing positions are reported to be vacant, with staff members often holding part-time positions, and around 25 % of the staff were not gerontology trained [9]. Immigrant nurses, often unfamiliar with the native country’s culture and history, can comprise up to 43 % of the staff [9]. Collectively, these factors may contribute to the marginalization of aged care homes as appropriate clinical placements for nursing students [9]. Yet, older people living in aged care homes have complex, but stable, nursing care needs, thereby rendering these settings as potentially ideal learning environments for first-year nursing students.

The predominant clinical placement model for nursing education in Norway and elsewhere in Europe is the preceptorship model [7.] In this model, students work with staff and receive mentoring from a registered nurse (RN) and follow up by a university-based nurse teacher [7, 10]. The teacher focuses on the relationship between the RN mentor and the student, supporting the integration of theoretical and practical learning. The teacher is responsible for coordinating the students’ learning, acting as a liaison rather than getting involved with hands-on patient care [10]. Hence, contact with and support from these teachers is important for first-year students. This support includes opportunities for critical reflection with peers to provide deeper learning about practice [11, 12].

Given the limited numbers of professional nurses or RNs working in aged care homes, the role of the nurse teacher in student learning is significant. For clinical placements, the qualities of the nurse teacher should enhance student learning [13, 14]. Nurse teachers should, therefore, be able to form positive relationships with students [15], meet them regularly [10], and be familiar with the nursing care in the placement setting [16]. In one study, differences in learning in an aged care home were attributed to the facilitation styles of nurse teachers [17]. How nurse teachers perceive their work may assist with further developing the nurse teacher role in the aged care home setting.

Placement in aged care homes is further complicated by limited access to experienced gerontology nurse teachers. Nurse teachers with backgrounds in acute care may be less interested in the care of older people and can bring negative attitudes into the aged care home setting [14]. Within universities, first-year student education is often relegated to the most junior academic staff, who may have limited teaching qualifications and may experience stress and role confusion, compromising their ability to effectively support students [18, 19]. The limited number of RNs to serve as mentors in aged care homes, combined with the lack of experienced gerontology nurse teachers generally, poses significant challenges to quality learning in aged care home placements. 

Globalization and migration have enhanced the internationalization of labour and education, leading to an increase in culturally and linguistically diverse students in internationally orientated nursing degree programs [20]. Over the last decades a sharp increase in the international mobility of students has been seen in Norway as well as globally [21]. Cultural diversity in nursing programs is perceived as an asset providing enriching experiences [20]. However, the greatest challenge for culturally and linguistically diverse students has been associated with clinical education [22], where students’ language proficiency influences the successful completion of clinical placements [23]. In a systematic review, one identified barrier to learning in clinical placement by culturally and linguistically diverse healthcare students was reported as experiences with university support and instructions [22].

A survey of Norwegian nursing students on placement in nursing homes found that students viewed the clinical learning environment more negatively than hospital placements on nearly all dimensions [24] as assessed by the Norwegian version of the Clinical Learning Environment, Supervision and Nurse Teacher evaluation scale [25]. The negatively reviewed dimensions included pedagogical atmosphere, nursing care and learning situations, content of the supervisory relationship and leadership style on the ward.

Despite an emerging body of research into how students learn and experience learning in aged care home placements, few investigations have examined faculty perceptions regarding implementing student placements in aged care homes. The reliance on aged care staff mentors with limited gerontological expertise, the complexity of teaching strategies within the preceptorship clinical education model, and the need for collaboration between mentors, students and nurse teachers suggest that further investigation is required. The aim of this study was to explore faculty staff perspectives on the challenges associated with providing effective clinical education in aged care homes to first-year student nurses.

## Methods

### Design

This study adopted an exploratory constructivist approach to qualitative interpretive inquiry. A constructivist approach assumes a relativistic ontology, acknowledging multiple realities, and a subjectivist epistemology, in which understanding is co-created by the participant and the researcher [26]. In-depth interviews [27] were used to explore faculty staff perceptions of the challenges associated with providing clinical education in aged care homes. In-depth interviewing is a research technique comprising intensive individual interviews with a small number of respondents in order to explore a new issue in detail [27]. The Standards for Reporting Qualitative Research (SRQR) guidelines were used [28].

### Setting and participants

The study setting comprised six nursing programs across three universities in Norway. These universities were purposively selected and predefined prior to data collection to provide variation [29] in terms of geographical area (rural/metropolitan), institutional size of the nursing programs (from 100 to 300 or more students), and institutional ranking. Institutional ranking is a national process used by the Norwegian higher education sector to monitor student experiences [30]. Both low- and high-ranking universities were included.

Recruitment was based on purposive criterion-based sampling [31]. Nurse program leaders, course leaders and practice coordinators were invited to participate. Nurse program leaders are responsible for the quality of bachelor’s degree programs in nursing. Course leaders are responsible for the content, administration, and quality of the courses, including aged care home placements. Practice coordinators are educational administrators responsible for placing students and coordinating with municipalities and placement sites.

Prior to data collection, approval was obtained from each of the three participating universities. Invitations to participate were emailed to eligible staff with information about the study.

### Data collection

A semi-structured interview guide was developed based on the integration of the literature and input from a panel of student nurses (*n* = 3), nurse teachers (*n* = 2) and nurse mentors (*n* = 2) (see Supplementary File 1). This panel of end-users ensured that the data collection tool was validated and anchored in the experiences of these key stakeholders. The guide addressed topics such as collaboration between education and practice, learning environment, mentors, nurse teachers, quality assurance work, barriers, and improvement measures. The guide allowed flexibility in responses depending on the participants’ interests and experiences. The first and last authors [KAL, IA], experienced qualitative researchers with backgrounds in nursing and education, conducted the interviews. The interviews were conducted in the participants’ workplaces and lasted approximately 60 min. Each interview began by explaining the study, confidentiality and informed consent procedure. The data were collected between November 2018 and February 2019.

### Data analysis

All interviews were recorded using a digital recorder and transcribed verbatim by three of the co-authors [KAL, CF, IA]. The analytical approach followed Braun and Clarke’s [32] framework for thematic analyses, a flexible approach to analysing qualitative data that searches for themes or patterns. Each theme captures data that informs the research question and can assist with identifying a response pattern or meaning within the data set [32]. Hence, the thematic analysis was guided by the research aim and followed the six phases described by Braun and Clarke [32]: (1) becoming familiar with the data; (2) generating initial codes; (3) searching for themes; (4) reviewing themes; (5) defining and naming themes; and (6) producing the report.

Four of the authors [KAL, CF, KA, IA] independently read the interviews to become familiar with the transcripts. They summarized and shared their impressions with the research team and explored different perspectives on the data. The transcripts were initially coded by the first and last authors, who highlighted segments relevant to the research questions to delineate patterns. The coded data were then sorted into potential recurring themes and patterns by looking for perceived challenges. Four of the co-authors [KAL, CF, KA, IA] discussed and achieved consensus by reviewing, modifying and refining the themes and potential sub-themes of the pre-defined analytic concept (e.g., challenges to clinical education). Five themes were identified. Examples of the themes were compared across the data items (e.g., interviews) and the most illustrative were selected for each theme. To ensure confidentiality and mask institutional affiliation, number identifiers were randomly assigned to each participant, including practice coordinators (e.g., PC1-5), program leaders (e.g., PG1-4) and course leaders (e.g., CL1-6).

### Ethical considerations

 The study was approved by the Norwegian Centre for Research Data (2018/61,309 and 489,776) and exempted from ethical approval by the Norwegian Regional Committees for Medical and Health Research Ethics since no health information or patient data was collected. Participation was based on informed, voluntary, written consent. To protect the anonymity of the participants and the educational institutions, details on university demographics, institutional and participant characteristics are not included in the paper.

## Results

Of the 17 potential respondents invited to participate, 15 consented. The participants were nurse program leaders (*n* = 4), course leaders (*n* = 6) and practice coordinators (*n* = 5) across the three universities. The analyses identified five themes related to perceived challenges to clinical education in aged care home placements for first-year student nurses: (1) low staffing levels of registered nurses limit the capacity to effectively host students; (2) prevalence of part-time teachers can compromise the quality of student experiences; (3) tensions about required qualifications and competence of nurse teachers; (4) variation in learning assessment; and (5) lack of quality assurance. These themes are presented and discussed below.

### Theme 1: Low staffing levels of registered nurses limit the capacity to effectively host students

All participants mentioned that aged care homes were generally welcoming of students on placement. However, placement capacity was limited by the low numbers of RNs available as mentors:

The main challenge, as I see it, is capacity for placement, as the entire class is in aged care home placement simultaneously. There is also lower nurse coverage in aged care homes than in other placement sites (PC1).

Participants from one educational institution tried to resolve this problem by dividing the class into two groups with different clinical placement periods. At another institution, students were placed in home healthcare due to the lack of capacity in aged care homes. Many participants emphasized that regulations governing students’ clinical placement within primary care and aged care homes were less sufficient compared to placement regulations in specialized health care:

The municipalities are not obligated to take students on placements as the hospitals are by regulation. So, it is a bit unpredictable. Suddenly, just weeks before placement, we get notice from an aged care home that cannot take students on placement after all, due to sick leaves, shortages of nurses and so forth. And, what do we do then? (PL2)

All three educational institutions reported having formalized agreements with the municipalities with stated capacity for students on aged care home placements. However, some participants indicated discrepancies between the number of placement positions offered and the number of students stipulated in the agreements. Participants described constant changes and unpredictability regarding the number of placement positions that complicated the placement planning process. Reasons for the reduction in placement positions offered by aged care homes included shortage of RNs, lack of supervisory competence, lack of time for mentoring, and understaffing. Moreover, the shortage of RNs could lead to suboptimal placements:

There is lower nursing coverage in aged care homes than in other placement sites. We want to have RNs to supervise the students. But due to the shortage of RNs or sick leaves, students might be followed up by experienced auxiliary nurses for parts of their placement period. Students are not always happy about that (CL2).

A few participants questioned whether limited placement capacity would compromise placement quality:

Clinical education capacity in aged care home placements is a real challenge. It is difficult to talk about quality, because we are in a state of debt of gratitude towards the municipalities and the aged care home placement sites as they accept students for placements. So, we need to be constantly thankful towards the nursing home placement sites. It also is difficult to make demands and talk about quality. Talking about placement quality becomes a bit secondary (PL3).

Although most participants claimed they had to find alternative ways to maintain positive collaborations within the clinical setting, these solutions sometimes created new problems. For example, one practice coordinator emphasized that some aged care homes had the capacity and the willingness to accept students and provide effective clinical placements. However, these facilities were in rural and remote areas that were difficult for students and the nurse teacher to access:

A lot of rural municipalities would be pleased to have and take students on placement in aged care homes. So, I do wish there was a way to supervise the students remotely. We are not using all the available placement sites in aged care homes because they are too far away for the nurse teachers to follow up with the students during placement (PC3).

Each nurse teacher was responsible for 4–24 students, which placed a strain on the available pedagogical support and feedback provided to students:

It is challenging for the nurse teachers when they have a lot of students to follow up during the placement period. When a teacher is responsible for following up 24 students, it becomes challenging to keep track and provide individual supervision and feedback (CL3).

Participants did not identify an upper limit of students per nurse teacher. It was emphasized that a teacher’s availability, work plan and wishes determined the nurse teacher to student ratio. A course coordinator indicated that a nurse teacher could be responsible for students in several aged care homes, and travel should, therefore, be a factor in student allocations.

### Theme 2: Prevalence of part-time teachers can compromise the quality of students’ learning experiences

Most participants reported that a large number of nurse teachers providing clinical education for first-year student nurses in aged care home placements were part-time staff. Participants reported that clinical RNs were hired to assume an educational role and act as nurse teachers. Aged care home placements for first-year student nurses emerged as the placement with the highest proportion of part-time nurse teachers:

One of the biggest challenges we have concerning first-year students’ placement in aged care homes is the lack of continuity among the nurse teachers. We agree that it is important to give students a good placement experience in their first-year placement. However, at our institution, almost half of the teachers we use in aged care home placement are external part-time staff (PL1).

Most of the participants reported that management did not prioritize clinical education nor question the high usage of external nurse teachers:

Management knows we need to hire nurse teachers externally to carry out students’ placements. It is the management that does not secure enough resources [to prioritize] clinical education. Management tells us to call, call, call somebody you know that can be hired to oversee clinical education. Management is fully aware of the situation. But nothing happens or changes. It has been like this for years (CL4).

Moreover, several participants reported that part-time nurse teachers were often recruited based on faculty employee acquaintances. There appeared to be few formal competence requirements for the nurse teachers to provide clinical education aside from being a RN. For example:

We ask our colleagues if there is someone they know that could act as teachers in clinical education in aged care home placements. So, it is a bit random. We sure want them [the hired teachers] to have a master’s degree. But a lot of them only have a bachelor’s degree. We do not have any specific educational requirements concerning the teachers we hire for overseeing clinical placements (PL4).

Most participants stated they preferred to use internal nurse teachers because first-year students often are more vulnerable and in need of support. However, several participants stated that because of shortages of nursing faculty it was difficult to avoid hiring part-time nurse teachers:

It is cheaper to hire a teacher to conduct clinical education than one with higher qualifications. However, [achieving a high-quality education] is difficult when we have a large number of externally hired nurse teachers to carry out clinical practice education that is not part of our internal staff (PL1).

A few participants claimed that clinical education and placement follow-up were a lower priority for the nurse education institutions and that nurses who held doctorates were assigned mostly to advanced research and education. According to one practice coordinator, ‘clinical education is given a lower priority among staff than other responsibilities’ (PC5).

In addition, participants noted the lack of formal preparation and orientation of the hired nurse teachers prior to the placement period. The educational institutions varied in their hiring practices. One educational institution mentored its externally hired nurse teachers; participants at other institutions claimed this was an area in need of improvement at their institution. In summary, across the educational institutions, problems existed regarding the competence and continuity of the staff entrusted with the supervision of students on placements.

### Theme 3: Tensions about required qualifications and competencies of nurse teachers

Participants offered diverse views on the competence level of the nurse teachers. One participant considered clinical experience and expertise far more important than their level of academic degree:

You do not need to be an associate professor to carry out clinical education. Nursing is a practical profession. People who have spent time building competence within academia have not been practicing nursing for a long time. I think students would benefit more from having clinical nurses with hands-on knowledge and expertise from the clinical field as nurse teachers. I think they can do a really good job (CL5).

In this quotation, the clinical competence of the teacher is considered critical for student learning in the workplace. However, most participants agreed that the nurse teachers’ pedagogical competence and the RN mentors’ competencies in supervision and assessment were most salient. Consequently, participants across educational institutions reported offering free courses to strengthen RN mentors’ supervisory competencies, but found it difficult to get the RNs to participate in these courses due to lack of financial compensation and practicalities:

The aged care homes do not have the resources to send the RN mentors on courses that the university offers. The mentors don’t want to participate on their day off and if they don’t get compensation time for the course by their employee. So, I believe that it is more practical challenges than the mentors’ willingness to enhance their supervisory competencies (CL6).

Moreover, some participants proposed that students’ learning in clinical education needed to be emphasized and that the RN mentors’ pedagogical competencies needed to be improved beyond their supervisory skills:

I wish we had a system where the RN mentors learned more about workplace learning, learning in general and supervision. There is insufficient focus on learning during the student’s placements in aged care homes (PL2).

When talking about the RN mentors’ competencies and supervisory skills, a program leader at one of the educational institutions noted:

It is a requirement that you as a teacher have university teaching and pedagogical basis of competence. But that is not the same as competencies in supervision. We talk about that RN mentors should be required to have supervisory competencies. However, no one is taking about the same requirements concerning the nurse teachers following up the students on placement. And that is interesting (PL1).

### Theme 4: Variation in assessment of learning

Across the educational institutions, assessment of students’ performance and competence was inconsistent, with various assessment instruments being used. One institution used a pass or fail grading scale, another used a verbal scale and the third used a numerical scale. The participants reported a range of satisfaction with these assessment instruments. Some justified using a numerical grading scale, while others preferred the freedom of writing a narrative. Several participants, especially course and program leaders, claimed that valid and reliable assessment of students’ competence was challenging due to the differences in tasks and student readiness. Reported challenges related to language difficulties, scoring/assessment of learning outcomes, assessment criteria, the RN mentor’s competence in assessment, and interaction among the student, RN mentor and the nurse teacher during the assessment process. Assessment was negotiated between nurse teachers and mentors, with nurse teachers making the final decision about individual student performance. For example:

Much is up to the nurse teachers; they have the last word. The nurse teachers take control, lead, and make final decisions concerning whether the students have achieved their learning outcomes. … The mentors are the ones that sees [sic] how the student performs in the care of patients and how they perform and behave in the clinical setting. (PL4).

Difficulties with the language used in the competence assessment documents were proposed as a potential barrier for interaction and involvement from both the student and the RN mentors during the assessment discussions. The participants reported that students and mentors reported difficulty understanding the concepts used to describe student learning outcomes and attributed this to linguistic challenges. For example:

It is challenging when you have students with a foreign first language and Norwegian as a second language. The same goes for the mentors. Because some of the mentors can also be difficult to understand due to linguistics. If you have a student and a mentor that both have linguistic challenges paired together, than you can have a real challenge. (CL4).

At another institution, the course leader shared a similar opinion:

It is difficult with students with linguistic challenges who have not mastered communication with patients or staff. Then, there can be a lot of misunderstandings. This is something I find worrying (CL5).

### Theme 5: Lack of quality assurance

 Few participants were familiar with goal-oriented efforts directed towards ensuring quality in clinical education in aged care home placements. Goal orientation existed at a faculty level, but not in terms of clinical education or placement quality:

We do not have a specific strategy when it comes to improving or ensuring placement quality – we do not (PL1).

A course leader at another educational institution concurred:

When it comes to quality in clinical education and aged care home placements, we don’t do much except provide supervisory courses to enhance the mentor’s competencies. Except for that we don’t do very much (CL3).

One participant reported that their institution monitored its own performance in clinical education and placement quality through self-reports from students, teachers, or the practice field (e.g., the RN mentors or placement sites). Some educational institutions collected students’ course evaluations, which included clinical education based on standardized course evaluations. However, several participants emphasized that evaluations were only randomly followed up. The responsibility for monitoring placement quality was seen as relying on the individual nurse teacher and his or her initiative to conduct evaluations after the placement period:

It is up to each individual teacher. Or, frankly, it is written in the instruction contract that the teacher should conduct an evaluation meeting with the stakeholders in the aftermath of placement. However, I must admit that I do not have full control over it, if they [evaluation meetings] are conducted and if so, who participates in these meetings, if it is with the RN mentors or with the management team at the aged care home. No, I strictly don’t know (PL1).

Moreover, several participants reported that if the nurse teachers conducted evaluations on their own initiative, these data were not followed up by the educational institutions and used for systematic quality improvements. For example:

If we gather information based on placement experiences from the various stakeholders, it will generate a huge amount of data. Evaluations commit. If we gather all these placement experiences and evaluations, we need to do something about them, follow them up in some way. We get struck with a lot of information without having a plan to proceed with it. A lot of times I feel that we gather a lot of information without knowing what to do with it and deal with it (CL2).

Most participants described the effort to assure the quality of clinical education as insufficient:

We do not conduct evaluation meetings with practice or with students in the aftermath of placement. We don’t. But it is something we probably should do and improve (PL3).

## Discussion

The findings from this study suggest at least two key challenges in providing effective first-year student clinical placements in aged care homes: (1) limited access for student placements; and (2) lack of qualified nurse teachers and available RN mentors. Other challenges pertain to the reliability and validity of assessment practices and the lack of a process for improving the quality of nursing student education from within the educational institutions.

Because the clinical placement model in aged care homes requires students to work with qualified staff [10], the capacity to host students is limited by the low number of RNs, understaffing and reliance on agency staff. Some placements were cancelled at short notice. Thus, the findings suggest that due to limited capacity, aged care homes may be less than optimal clinical placements.

Norway has more than 950 aged care homes [9], which should ensure an adequate number of placement opportunities. A case study of selected Norwegian nursing homes found variations in nurse competence and staffing [33], with consequences for students’ access to RNs. In the current regulatory environment, this variability complicates the formation of collaborative legal agreements between the higher education institution and each aged care home. These agreements may also differ when accounting for the unique capacities of each municipality and their geriatric facilities. Making and monitoring these arrangements is labour-intensive, which may create an impediment to aged care home placements for nursing students.

In Australia, guidelines for collaborative agreements have been developed [34]. Creating such guidelines as part of a broader policy program around quality clinical supervision for students in aged care homes and other health services may enhance access to aged care homes for student placements. However, these agreements must specify minimum staffing levels to ensure adequate student learning. Further research into the minimum levels of staffing required to support student learning in the aged care home is required. In Norway, as in most EU countries, no specific educational requirements or national standards guide the quality of clinical practice placements and mentoring practices [35]. However, in the UK, national standards for clinical education have existed for many years [36].

The lack of qualified teachers in aged care homes is manifested as a high proportion of part-time nurse teachers and staff with limited geriatric qualifications and competence. Moreover, there was strong agreement among participants that part-time nurse teachers did not provide consistency for first-year students, as they are not involved in theoretical teaching on campus prior to placement. Further, given the shortage of RNs available to mentor and support student nurses’ learning in aged care homes, support from nurse teachers may become even more important. Indeed, a study by Skaalvik et al. [37] found that students evaluated the role of the nurse teacher as more important in their first year than in their final year of study. Furthermore, the use of part-time nurse teachers in aged care homes required a high resource investment to prepare teachers for their roles and often lacked a systematic approach, leading to high variability in quality. Methods for preparing teachers to work in the aged care setting require further development, including online options possibly supported by a community of practice model for peer support and development of shared practices.

Other approaches to clinical education in aged care homes require further investigation. Due to budget cuts and increasing constraints, educational leaders may commit to long-term partnerships with one or a small group of aged care homes [38]. Having nurse teachers consistently assigned to the same aged care facility is essential for developing and strengthening their collaborative relationship [38]. In emerging models, nurse teachers can be jointly funded by the facility and the institution of higher education partners. Between student placements, these teachers could offer education and training in student supervision for RNs and collaborate with staff to identify and develop learning activities that are appropriate for students’ learning objectives. This model has been used in the Student Nurse Led Ward model of clinical education in aged care homes with some success [39].

A limited amount of teachers in aged care homes possess geriatric qualifications and competencies. In the United States, a group of gerontology nurses has developed national competency standards for gerontological nurse educators, which are endorsed by the National Hartford Center of Gerontological Nursing Excellence [40]. Further research into the applicability of these competencies to the nurse teacher role is aged care homes is warranted. For sustainable and high-quality clinical education, future research should critically explore and extend our understanding of the relationship between nurse teachers’ clinical expertise, competence, pedagogical skills set and students’ learning outcomes.

Lack of pedagogical competence and supervisory skills among RN mentors (e.g., clinical nurses) who supervise students in aged care homes reflects a frequently mentioned challenge related to supervision of students in aged care facilities [24]. Developing educational interventions such as web-based educational support and peer networks for RN mentors may also provide a method for improving their pedagogical knowledge and mentorship practices [41].

Another notable challenge in providing clinical education in aged care homes pertained to consistent, reliable, and valid assessment practices. This challenge is not unique to aged care homes or to Norway. Research into clinical nurse and academic perspectives on student clinical assessment in Singapore suggests that the lack of a valid and reliable assessment tool, limited mentor competence in assessment, and limited academic-clinical collaboration were common issues [42]. A systematic review of student competence assessment found that the use of a valid and reliable assessment tool, with clearly identified criteria, and continued education and support for mentors are critical to quality learning [43]. Moreover, study findings indicate that nurse teachers play a dominant role during the assessment process due to a lack of engagement from RN mentors. Given the homogeneity in aged care home populations, there is an opportunity to develop an international collaborative into learning and assessment in aged care homes, possibly starting with establishing standards for first-year nursing students.

However, language difficulties associated with an internationally diverse workforce and student population appeared to have a profound effect on students’ assessment in the aged care home context, contributing to reduced clarity about performance expectations. In this study linguistic challenges among students and RN mentors were reported to constrain student competence assessment. The importance of workforce diversity within healthcare systems to reduce health care disparities has been emphasized [44]. However, the effects of linguistically diverse RN mentors on mentorship and assessment practices, as well as on student learning in aged care placement, are not well established. Further research into the emerging educational challenges associated with international diversity is required.

The final challenge was the lack of a systematic process for improving the quality of nursing students’ clinical education, especially in aged care homes. Some institutions collected information from clinical placements, but the volume of data produced was difficult to review due to the academic staff’s limited resources. For those collecting student experience data, the great variation in placement experiences is consistent with other research conducted in Norway [45]. For a richer and more cohesive understanding of the aged care home as a clinical placement, evaluations and feedback from RN mentors, the placement host, nurse teachers and student nurses are recommended. Again, an international collaborative is required to develop standards for the quality of educational experiences in the aged care home setting.

Clinical placements in aged care homes may not be sufficient preparation for graduate nurses who will be working with an older population. Lane and Hirst [4] recommend, at a minimum, a carefully constructed curriculum incorporating gerontological theories and placement experience in aged care homes. McSharry and colleagues [46] suggest that lecturers have a responsibility to bring student experiences from aged care homes into the classroom for analysis and further learning. As curricula are designed with more geriatric content, comprehensive studies are required to investigate those educational strategies that improve student competence in geriatric care [3].

### Study limitations

This study has several limitations that merit consideration when interpreting the findings. First, this study was based on a relatively small sample conducted within a Norwegian context, which restricts the transferability of the findings. Nevertheless, the findings and issues raised are relevant in a national and international context. Sample size and data saturation in qualitative research has been subject to enduring discussions due to a variety of conceptual understandings [47]. In this study, sample size was defined prior to data collection according to the purposeful criterion-based sampling strategy [31] and the predefined study settings. The sample was highly specific for the aim of the study and the interview dialogue was strong, which enhances information power [48]. The concept of information power is related to saturation, and indicates that high sufficiency of information within a sample deemed relevant for the study aim requires a lower number of participants [48]. Furthermore, a high degree of consensus emerged during data analysis, in which themes were replicated across the data set and deemed sufficient to satisfy the exploratory nature of this in-depth study [49].

Furthermore, potential biases should be acknowledged since the data collection and analysis were conducted by researchers with a background in nurse education, which entails a prior understanding of the context. To control for research bias, we applied triangulation during data analysis. Four of the authors participated in data analysis and reflected upon the findings, which provided a basis for checking interpretations [50]. The analysis was not reviewed by the participants (i.e., member checking of transcripts and interpretations), which could have been conducted to verify the findings [50].

### The study implications on practice

While student placements in aged care homes are important for student learning, the variability of these homes in terms of staffing, combined with the limited preparation of nurse teachers to work in this setting, renders high quality learning serendipitous. A regulatory approach to including geriatric content and experience in undergraduate nursing curricula is long overdue. While a first-year placement offers opportunities to learn about the essence of nursing, opportunities for second-year students to learn more about the clinical presentations of complex comorbidities in this setting have yet to be explored. As the population over 80 years of age continues to grow, the need for nurses in aged care homes will continue to rise. To increase the number of qualified and competent nurses in aged care homes, further research into the benefits of placements for second- and third-year students is required.

Overall, there is an urgent need for national, and possibly international, standards for gerontological nursing education in undergraduate nursing programs, including standards and guidelines to support students’ placements. As national governments examine and develop policies to address the burgeoning health issues associated with an ageing population, investment in geriatric nursing knowledge as a generalist competency through educational standards is recommended.

## Conclusions

The world population is ageing, and as people live longer, many will experience chronic multiple morbidities. A nursing workforce prepared to work with older people is critical. Aged care homes offer a unique opportunity for first-year undergraduate nursing students to learn more about older people and chronic diseases and to develop skills to communicate and assist them with daily living activities. However, the sector’s lack of maturity in providing educational experiences, particularly in relation to the quality of staff, is a barrier to effective placements. While the health services have a history of hosting students and are widely accepted as suitable environments for student learning, optimal learning environments are not yet fully developed in aged care homes. Providing such an environment will require industry regulation around staffing for student supervision and collaboration between the aged care sector and universities to set standards for nurse teachers and training.

This study investigated challenges to clinical education in aged care homes as perceived and experienced by faculty staff. The research provides insight into the many impediments to students’ clinical education in these settings, which limit the quality of placement experiences as well as the maximization of learning potential. Hence, we propose that targeted efforts are warranted to enhance student nurses’ clinical education in aged care homes.

In conclusion, we propose that nurse education institutions, in partnership with aged care homes, must invest in clinical education. Collaboratively, the academic-practice institutions must lead changes to improve the quality of students experience in aged care homes by evaluating their practices and contributions in clinical education and by engaging with government officials in health and aging as well as national nursing bodies. Evaluations should be based on indicators appropriate for the aged care home setting that integrate a multiple stakeholder perspective. Moreover, the findings call attention to the need for national regulations and incentives and international research and development collaboratives that support quality of learning in aged care home placements. Taken together, these measures may enhance placements that stimulate and maintain students’ interest in the care of older people.

## Supplementary Information

Additional file 1: **Supplementary File 1.** Interview Guide.

## Data Availability

The data sets used and analysed for the current study are available from the corresponding author on reasonable request.

## Abbreviations

SRQR: Standards for Reporting Qualitative Research
RN: Registered nurse
