# Supplementary material for: Enhancing student nurses’ clinical education in aged care homes: a qualitative study of challenges perceived by faculty staff
Source: BMC Nurs. 2021 Jun 26;20:111. doi: 10.1186/s12912-021-00632-0 (PMC8235807; doi:10.1186/s12912-021-00632-0)
Supplement: Supplementary file 1 — Interview Guide. [file 12912_2021_632_MOESM1_ESM.docx]

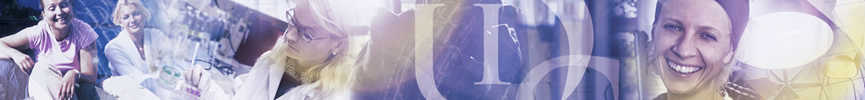


Interview Guide

**Opening questions:
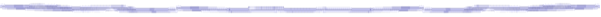
**

- What are your responsibilities as practice coordinator/course leader/program leader?
- For how long have you had this position?
- What are your specific tasks/responsibility concerning clinical placement in nursing homes?

**Collaboration between the university nursing school and nursing home practice sites**


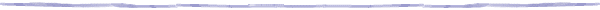


- How will you describe the nursing school’s collaboration with the nursing home practice sites?
  - What in your opinion works well?
  - What in your opinion works less well? (challenges/barriers)
- What do you and your organization do promote/facilitate a good relationship between the nursing school and the nursing home practice sites) (measures)
- How will you describe barriers and success criteria for a good relationship/good collaboration?

**Nurse teachers
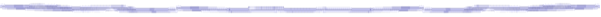
**

- Describe characteristics of the nurse teachers that is responsible for overseeing first year student nurses on placement in nursing homes.
  - How are they recruited?
  - What kind of formal competence requirements exist?
  - What kind of measures are in place to prepare nurse teachers/educators for their responsibilities prior to the student’s placement?
- In your opinion are there any challenges, barriers and or improvement measures related to issues concerning the nurse teachers responsible for overseeing first- year students on placement
  - If so, what would they be?

**Learning environment in nursing homes
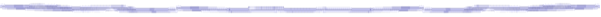
**

- In your opinion what characterizes a safe and good learning environment for first-year students in nursing home placements?
- What do the nursing school do to facilitate/enhance a safe and enriched/good learning environment for first-year students in nursing home placement
- How do you work to develop the students learning objectives for clinical placement in nursing homes?
  - Who are involved?
  - How often and how are the learning objectives evaluated – and who participate in the evaluation
- How are the students learning objectives communicated to the students, practice field (e.g. RN nurse mentors)?

**Registered nurse mentors**


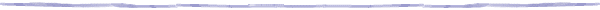


- How will you describe the RN mentor’s overall competence in student supervision?
- What do the nursing school do to enhance the nurse mentor’s supervisory competence?
  - What kind of measures?
  - The use/ and utility of those measures
  - What is the feedback from the practice field?
- How does the nurse education system prepare RN mentors prior to the student’s placement in nursing homes? (challenges, barriers, improvement measures)
- How are the Nurse mentors followed up by the nurse education system during the student’s placement period (pre-during and post-placement)?

**Assessment of students during placement**

**
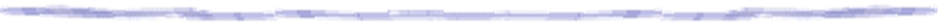
**

- How is assessment of first-year student nurses organized (both formative and summative assessment)
- What kind of assessment form do you use and what are the assessment criteria?
- What are your experiences, and perceptions of the assessment process and the form applied?
  - Strengths, weaknesses ect
  - Challenges, barriers, improvement measure ect
  - What is the pedagogical rationale for the assessment form applied?

**Quality assurance work**

**
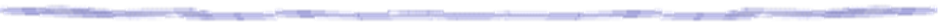
**

- From your perspective what characterizes quality in clinical placement in nursing homes?
- What do the nurse education system do to enhance quality in the students clinical practice placements in nursing homes (proactive, reactive measure)
- Describe how the students practice placement period is evaluated?
- How is information/outcome of learn and improve?
- How do you know what areas you are good at and what areas you need to improve?
- What are your thoughts on variability in supervision and assessment of students in clinical practice education in nursing homes?
- What to you consider as necessary and adverse variation?
- What in your opinion needs to be done to enhance nursing homes as enriched/high quality learning environments for student nurses?
  - Follow-up question: What are the challenges, barriers to achieve such improvements
- Imagine in a dream life: How would you envision student’s clinical placement in nursing homes - supervision, assessment ect.

**Closing remark:** According to a Norwegian Survey conducted by the Norwegian Nurses Organization

In 2018 only 9% of graduate students report that they could envision themselves working in a nursing home after graduation. What are your thoughts on that? -and in your opinion is there something the nursing school could do to enhance students view of nursing homes as a more attractive career choice

Is there anything else you wish to address or add before we round off?

**Prompts:**

If a question is answered with a simple yes or no, ask the participant to explain further.

For example, you can ask “why” or “why not”?

Other generic prompts include: ““Can you tell me more about this?”; “Can you explain this

further / expand on this?” and “What do you mean when you say ____?
